# Real-world experience with RESCUE BT2 tirofiban protocol versus standard care for select patients with acute ischemic stroke at a United States comprehensive stroke center: a single-center retrospective study

**DOI:** 10.3389/fneur.2026.1768603

**Published:** 2026-07-22

**Authors:** Supreet Kaur, Kevin Dang, Diane McLaughlin, Kassu Mehari Beyene, Alexander Nicholas, J. Tyler Haller, Bryan Franzen, Vishal Harnoor, Tracie Schroeder, Alisha Qaiser, Siddhant Arora, Jennifer Smitt, Abbi Heater, Brandon May, Josiah Chang, David Wang

**Affiliations:** 1 Department of Neurology, Barrow Neurological Institute, Phoenix, AZ, United States; 2 Department of Neurosurgery, Barrow Neurological Institute, Phoenix, AZ, United States; 3 Department of Pharmacy, St. Joseph’s Hospital and Medical Center, Phoenix, AZ, United States; 4 Department of Neurology, Duke University School of Medicine, Durham, NC, United States; 5 Department of Neurology, Honor Health: Arizona State University John Shufeldt School of Medicine and Medical Engineering, Tempe, AZ, United States

**Keywords:** acute ischemic stroke, early neurological deterioration, standard care, tirofiban, United States

## Abstract

**Background:**

Tirofiban, a glycoprotein IIb/IIIa receptor inhibitor, has shown promise in acute ischemic stroke (AIS) without medium- or large-vessel occlusion. The RESCUE BT2 trial demonstrated improved functional outcomes with IV tirofiban versus aspirin, but was conducted predominantly in Han Chinese populations, limiting generalizability to U.S. practice. This study evaluated the safety, operational feasibility, and exploratory clinical associations of a RESCUE BT2-based tirofiban protocol at a U.S. comprehensive stroke center.

**Methods:**

This retrospective single-center cohort study compared patients treated with tirofiban per RESCUE BT2 protocol (Nov 2023–June 2025) with a historical standard care cohort meeting similar eligibility criteria (Jan 2021–Oct 2023). Primary outcomes focused on safety, including any intracranial hemorrhage (ICH), symptomatic ICH, extracranial bleeding, thrombocytopenia, and mortality. Exploratory clinical outcomes included NIHSS trajectories, discharge modified Rankin Scale (mRS), discharge disposition, hospital length of stay, and available 90-day mRS. Multivariate regression models adjusted for baseline severity, early therapies, and neurological severity at the clinical decision point. Propensity score matching and/or multiple imputation were conducted as sensitivity analyses, and the results were interpreted as exploratory.

**Results:**

Seventy-four patients were included (32 tirofiban, 42 standard care). No patients in the tirofiban group developed symptomatic or asymptomatic ICH, and no thrombocytopenia was observed. Extracranial bleeding occurred in 3 patients (9.4%). In adjusted exploratory analyses, tirofiban was associated with lower discharge NIHSS (*β* = −2.199; 95% CI, −3.850 to −0.548; *p* = 0.011), greater NIHSS improvement from the decision point to discharge (*β* = 2.815; 95% CI, 1.173–4.456; *p* = 0.001), and a favorable shift in discharge mRS (OR 0.274; 95% CI, 0.087–0.86; *p* = 0.027). In propensity-matched analysis, the direction of effect for discharge mRS was consistent but did not reach statistical significance. 90-day mRS analyses were limited by substantial differential missingness; complete-case and propensity-matched analyses were non-significant, and multiple-imputation results were sensitive to model assumptions.

**Conclusion:**

Implementation of a RESCUE BT2-based IV tirofiban protocol in a U.S. comprehensive stroke center was feasible and demonstrated a favorable safety profile in carefully selected AIS patients. Exploratory associations with improved neurological trajectories warrant prospective evaluation in larger multicenter studies.

## Introduction

Stroke is a major cause of death and disability worldwide (1). While interventions such as intravenous thrombolysis (IVT) and endovascular thrombectomy (EVT) have significantly improved acute ischemic stroke (AIS) outcomes over the past three decades, fewer than 5 % of patients actually receive these treatments (2, 3).

Moreover, a significant concern following AIS is early neurological deterioration (END). END occurs in 15–40% of patients, most often due to stroke progression, and is a major predictor of poor outcomes (4–7). Despite ongoing progress, current American Heart Association/American Stroke Association guidelines do not recommend any targeted therapy for END beyond standard antiplatelet regimens, revealing a possible therapeutic gap (2). This is particularly relevant for patients presenting outside the window for IVT, are ineligible for IVT, or worsen despite guideline-directed treatment.

Accelerated platelet aggregation and microthrombus formation in the ischemic microcirculation are likely major contributors to END, although multiple mechanisms may be involved (8–19). Tirofiban, a reversible glycoprotein IIb/IIIa inhibitor with rapid onset, short half-life, and predictable reversibility, provides potent inhibition of the final common pathway of platelet aggregation (20, 21).

While recent randomized controlled trials comparing tirofiban to aspirin have yielded mixed outcomes, they generally show positive, though variable, results concerning early neurological improvement and reduced early neurological deterioration. Furthermore, these trials indicate better 90-day functional outcomes without a significant increase in symptomatic intracerebral hemorrhage (sICH) (22–31).

Despite encouraging data, substantial limitations hinder direct application to stroke care in the United States (U.S.). Nearly all modern trials, including TREND, RESCUE BT2, ESCAPIST, were conducted in the predominantly Han Chinese population where underlying stroke mechanisms, particularly the high prevalence of intracranial atherosclerosis, differs significantly from those in the heterogeneous U.S. population (23–30). Additionally, questions regarding generalizability arise when considering differences in workflow, antiplatelet selection, imaging, and post-stroke management. To our knowledge, no prior study has evaluated the safety, operational integration, or early clinical effects of tirofiban within the U.S. AIS population.

Based on the promising results of prior studies, our institution implemented a protocol for intravenous (IV) tirofiban use in carefully selected patients with AIS modeled after the RESCUE BT2 (Efficacy and Safety of Tirofiban Compared with Aspirin in the Treatment of Acute Ischemic Stroke) trial (28). The primary objective of this study was to evaluate the safety and operational feasibility of implementing this protocol within a contemporary U.S. comprehensive stroke center. Secondary exploratory objectives included comparison of neurological and discharge functional outcomes with a historical standard care cohort meeting similar eligibility criteria.

## Methods

### Study design

Following institutional review board approval, a retrospective, single-center study was performed comparing Tirofiban to standard care in patients with AIS who met the inclusion criteria of the RESCUE BT2 randomized controlled trial (detailed below). Given the retrospective nature of the study, informed consent was not obtained from the patients. The study adhered to the STROBE guidelines, the Health Insurance Portability and Accountability Act, and the Declaration of Helsinki (32, 33).

### Setting

The study included patients with AIS treated with tirofiban at our comprehensive stroke center from November 2023 to June 2025. The historical comparison group included patients with AIS admitted from January 2021 to October 2023 at our institution.

### Study cohort

#### Tirofiban group

Tirofiban protocol was implemented at our comprehensive stroke center in November 2023. Patients were identified using IRB-approved, prospectively-collected quality improvement tirofiban registry. Consistent with the RESCUE BT2 inclusion and exclusion criteria (28), treatment was offered to patients with AIS who had a National Institute of Health Stroke Scale (NIHSS) of five or more, at least one moderate to severely weak limb, no evidence of large or medium vessel occlusion, and met one of the following criteria:

- Ineligible for reperfusion treatment and within 24 h of stroke onset.
- Ineligible for reperfusion treatment and showed END 24–96 h after stroke onset, defined as an increase in the NIHSS score by at least 2 points.
- Received IVT followed by END, defined as an increase in NIHSS by at least 4 points.
- Received IVT followed by no improvement, defined as a decrease in NIHSS by 2 points or less between 4 and 24 h after IVT.

Exclusion criteria included any surgical intervention, including endovascular therapy, carotid intervention, decompressive hemicraniectomy, external ventricular drain; patients with intracranial hemorrhage (ICH) on baseline imaging; any definite source of cardiac embolism.

The decision to use tirofiban was at the discretion of the treating physician. A screening log for patients who may have been eligible but not offered Tirofiban was not kept.

#### Standard group

The historical comparison cohort was identified through our prospectively-collected stroke center quality database. Patients were excluded if they underwent any procedures (endovascular thrombectomy, carotid intervention, or decompressive hemicraniectomy), had hemorrhagic transformation on subsequent imaging, or if their NIHSS score consistently stayed less than five from admission to 24 h and discharge. The remaining patients were individually reviewed by two raters blinded to final discharge outcomes (SK and KD). Patients who met the RESCUE BT2 inclusion criteria were finally included in the study.

### Treatment

All patients admitted with AIS at our institution receive American Heart Association/American Stroke Association guidelines recommended treatment (2). Imaging, typically computed tomography (CT) Head and CT angiography of head and neck, is obtained for all patients at admission. Magnetic resonance imaging is subsequently obtained for all patients, unless contraindicated or not tolerated.

#### Tirofiban group

IV tirofiban was given at a dose of 0.4 μg/ kg/ min for 30 min, followed by a continuous infusion of 0.1 μg/kg/min for up to 48 h (21, 28). Tirofiban order set and Pharmacy oversight were maintained to ensure appropriate dosing and timing.

During the tirofiban infusion, other antithrombotic medications were held, though deep vein thromboprophylaxis was maintained. Following the completion of the infusion, standard antithrombotic regimens were resumed if no hemorrhagic conversion was noted on the follow- up imaging.

Depending on their associated comorbidities and specific patient care needs, patients were managed in either the neuro intensive care unit (ICU) or stroke floor unit with continuous telemetry monitoring.

Protocol-directed neuroimaging (typically noncontrast CT head) was obtained before tirofiban initiation and following completion of the infusion, which generally occurred within 48 h of treatment initiation, to evaluate for hemorrhagic transformation.

### Data collection

Data regarding patient demographics, admission NIHSS score, use of IVT, hospital length of stay, and discharge disposition were obtained from our prospectively-maintained stroke center quality database. A retrospective chart review was also conducted using the hospital’s electronic medical record system (Cerner) to gather additional information, including: vascular risk factors, antiplatelet medication use before admission, medication administration records, blood pressure measurements (at admission and at 24 h or time of END), NIHSS scores (at 24 h or time of END), discharge NIHSS score, and modified Rankin Scale (mRS) score at discharge and 90-days.

The premorbid modified Rankin Scale (mRS) score was obtained from the physical therapy initial evaluation note. The treating team, all of whom are certified in administering the NIHSS and mRS, documented both the NIHSS and mRS scores.

Mechanism of stroke was determined by a stroke neurologist (BF) who individually reviewed each chart and was blinded to the discharge outcomes. The Trial of Org 10,172 in Acute Stroke Treatment (TOAST) criteria was applied to classify the stroke mechanism (34).

Cerebral infarction location, laterality, and hemorrhagic conversion were captured after independent review of imaging by a stroke neurologist and two neurovascular fellows (SK, AQ, and SA) who were blinded to patient outcomes.

All data were de-identified and stored within RedCap for analysis.

### Outcome measures

Primary outcomes focused on safety. These included any ICH identified on follow-up neuroimaging approximately 48 h after treatment initiation, sICH, extracranial bleeding, development of thrombocytopenia (platelet count below the lower limit of normal, i.e., 150,000/microliter for adults); and in-hospital mortality (mRS = 6). The primary definition of sICH was based on ECASS II (35) criteria. For comparability with prior stroke literature, sICH was additionally assessed using NINDS (36) and SITS-MOST (37) definitions.

Exploratory clinical outcomes included NIHSS (scores range from 0 to 42, with higher scores indicating a more severe deficit) (38), and modified Rankin Scale (mRS; which ranges from 0 to 6, with a score of 0 indicating no disability and higher scores indicating more severe disability) (39) at discharge, length of stay, and discharge disposition.

### Statistical analysis

Given the retrospective exploratory design, no prior sample size calculation was performed. Participant characteristics and study variables were summarized using descriptive statistics. Categorical variables were presented as frequencies (*n*) and percentages (%) and were compared between treatment groups using chi-square or Fisher’s exact test, as appropriate. Continuous or ordinal variables were reported as the median with interquartile range (IQR) or the mean with standard deviation (SD), and were compared using the Mann–Whitney U test or Welch’s two-sample t-test.

Regression was used to quantify the association between treatment assignment and discharge outcomes while adjusting for differences in baseline severity/early therapies and neurological severity prior to tirofiban treatment between groups. Unadjusted models included treatment only. Baseline-adjusted models included treatment plus covariates (age, sex, NIHSS at admission, prior stroke, diabetes mellitus, and reperfusion therapies (IVT/EVT)) to account for baseline severity and key early therapies. Decision-point-adjusted models included all baseline-adjusted covariates plus NIHSS at END or 24 h to additionally account for neurological severity immediately prior to tirofiban administration.

Crude odds ratios were estimated using univariable logistic regression. Adjusted odds ratios were estimated using multivariable logistic regression. For continuous outcomes, estimates were obtained using linear regression. For NIHSS-derived outcomes, we did not adjust for NIHSS measures that are embedded in the outcome (e.g., admission NIHSS when modeling change from admission-to-discharge) to avoid redundant adjustment and improve interpretability of the treatment estimate.

As a sensitivity analysis addressing potential treatment-selection bias, propensity score matching was performed using age, sex, and premorbid mRS. Baseline balance before and after matching was assessed using standardized mean differences. The matched cohort included 27 patients in each treatment group. Outcomes were re-estimated in the matched cohort using the same unadjusted, baseline-adjusted, and decision-point adjusted model structure used in the exploratory clinical outcome analyses.

Analyses of 90-day mRS were limited by substantial differential missingness between treatment groups. Little’s MCAR test was performed to evaluate whether the missingness pattern was consistent with missing completely at random (MCAR) assumptions. Therefore, 90-day mRS was evaluated using complete-case analysis and additional sensitivity analyses with multiple imputation using 50 imputed datasets and propensity-score matching.

As an additional sensitivity analysis, exploratory mRS discharge and 90-day mRS models were repeated after excluding patients with documented protocol deviations. This analysis used the same model structure used in the primary exploratory analyses, including unadjusted, baseline-adjusted, and decision-point adjusted models. Findings were interpreted descriptively because the exclusion of protocol deviations further reduced the analyzable sample size.

## Results

### Baseline characteristics

**Figure 1:**
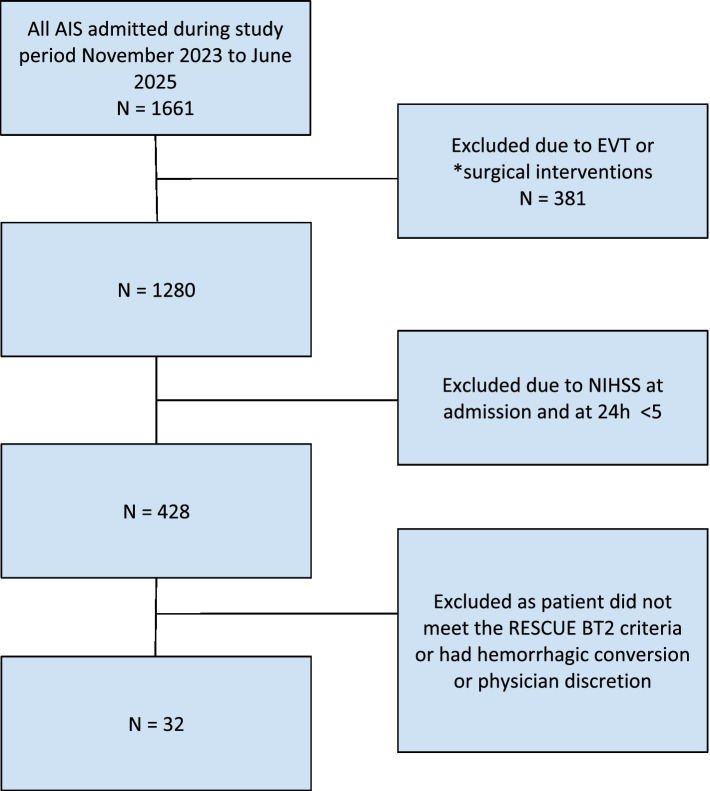
Inclusion/exclusion flow chart for tirofiban group. AIS, Acute ischemic stroke; EVT, endovascular thrombectomy; NIHSS, National Institutes of Health Stroke Scale. *Surgical interventions included carotid endarterectomy, carotid stenting, decompressive craniectomy and external ventricular drain placement.

**Figure 2:**
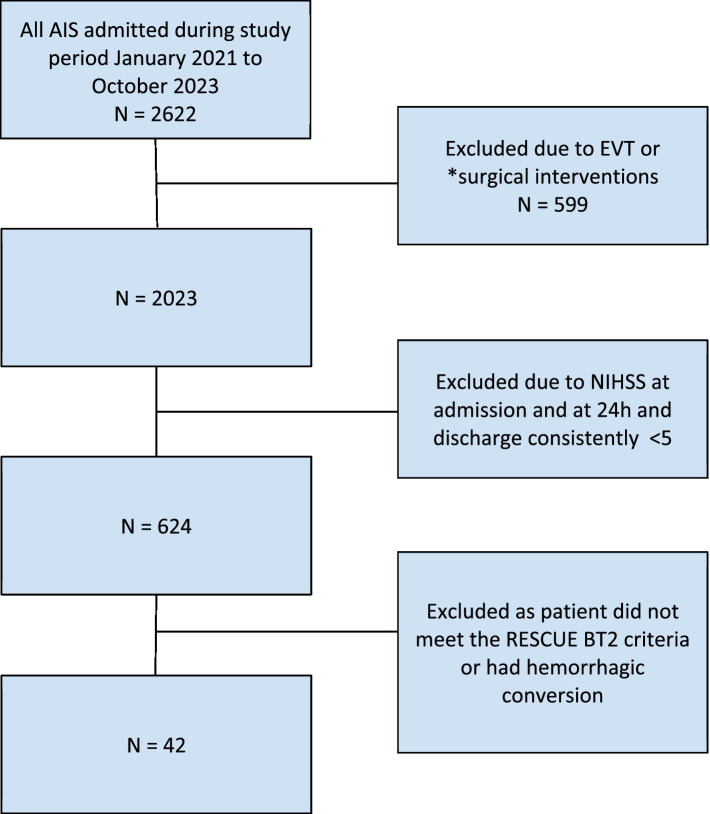
Inclusion/exclusion flow chart for standard care group. AIS, Acute ischemic stroke; EVT, endovascular thrombectomy; NIHSS, National Institutes of Health Stroke Scale. *Surgical interventions included carotid endarterectomy, carotid stenting, decompressive craniectomy and external ventricular drain placement.

Patient selection is outlined in the flowchart provided in Figures 1, 2.

The study population’s baseline characteristics are summarized in Table 1. The cohort was predominantly male (63.5%) and white (82.4%), with a mean age of 66 years. Vascular risk factors were highly prevalent, with nearly nine out of 10 patients (89.2%) presenting with both hypertension and hyperlipidemia. Additionally, a substantial portion of the cohort reported tobacco use (47.3%) and was categorized as obese (31.5%). Prior to admission, 35.1% of patients were already on antiplatelet therapy, most frequently aspirin.

**Table 1:** Baseline characteristics.

Baseline characteristics	Tirofiban	Standard care	Total	*p*-value
	*N* = 32	*N* = 42		
Age, mean (SD)	67.28 (10.23)	65.86 (10.37)	66.47 (10.26)	0.557
Male sex, *n* (%)	22 (68.8)	25 (59.5)	47 (63.5)	0.414
Race, *n* (%)				
White	24 (75)	37 (88.1)	61 (82.4)	0.496
Black	2 (6.2)	3 (7.1)	5 (6.8)	
American Indian	2 (6.2)	1 (2.4)	3 (4.1)	
Multiracial/other	2 (6.2)	1 (2.4)	3 (4.1)	
Asian	1 (3.1)	0 (0)	1 (1.4)	
Decline to state	1 (3.1)	0 (0)	1 (1.4)	
Vascular risk factors, *n* (%)				
Hypertension	30 (93.8)	36 (85.7)	66 (89.2)	0.453
Hyperlipdemia	30 (93.8)	36 (85.7)	66 (89.2)	0.453
Diabetes	5 (15.6)	29 (69)	34 (45.9)	**<0.001**
Coronary artery disease	4 (12.5)	7 (16.7)	11 (14.9)	0.747
Atrial fibrillation	4 (12.5)	1 (2.4)	5 (6.8)	0.159
Congestive heart failure	3 (9.4)	0 (0)	3 (4.1)	0.077
Prior stroke	4 (12.5)	15 (35.7)	19 (25.7)	**0.024**
Tobacco use	14 (43.8)	21 (50)	35 (47.3)	0.594
Obesity	11 (34.4)	12 (29.3)	23 (31.5)	0.641
Antiplatelet use prior to admission, *n* (%)	9 (28.1)	18 (42.9)	27 (36.5)	0.192
If yes, antiplatelet used?				
Aspirin	5/9 (55.6)	12/18 (66.7)	17/27 (63)	0.346
Clopidogrel	0/9 (0)	1/18 (5.6)	1/27 (3.7)	
Aspirin and Clopidogrel	2/9 (22.2)	5/18 (27.8)	7/27 (25.9)	
Aspirin and Cangrelor	1/9 (11.1)	0/18 (0)	1/27 (3.7)	
Apixaban	1/9 (11.1)	0/18 (0)	1/27 (3.7)	
Premorbid mRS, median (IQR)	0 (0, 0)	0 (0, 0.75)	0 (0, 0)	0.303
NIHSS at admission, median (IQR)	4.5 (2, 8)	5.5 (5, 7)	5 (4, 7.75)	0.146
SBP on admission (mmHg), mean (SD)	178.66 (39.44)	165.12 (29.11)	170.97 (34.38)	0.108
DBP on admission (mmHg), mean (SD)	99.53 (28.06)	89.45 (17.68)	93.81 (23.13)	0.081
Location of infarct, *n* (%)				
Cortical	5 (15.6)	11 (26.2)	16 (21.6)	0.274
Corona radiata/Periventricular	2 (6.3)	5 (11.9)	7 (9.5)	0.691
Basal Ganglia	0 (0)	2 (4.8)	2 (2.7)	0.502
Internal Capsule	2 (6.3)	6 (14.3)	8 (10.8)	0.453
Thalamus	1 (3.1)	1 (2.4)	2 (2.7)	1
Brainstem	10 (31.3)	6 (14.3)	16 (21.6)	0.079
Multiple areas	11 (34.5)	11 (26.2)	22 (29.7)	0.445
No cerebral infarction on MRI Brain	1 (3.1)	0 (0)	1 (1.4)	0.432
Laterality of infarct, *n* (%)				
Left	17 (53.1)	21 (50)	38 (51.3)	0.683
Right	8 (25)	18 (42.8)	26 (35.1)	0.133
Bilateral	6 (18.8)	3 (7.14)	9 (12.1)	0.156
Mechanism of infarct (TOAST classification, *n* (%))				
Small-vessel disease	17 (53.1)	26 (61.9)	43 (58.1)	0.061
Large-artery atherosclerosis	10 (31.3)	16 (38.1)	26 (35.1)	
Cardioembolism	3 (9.4)	0 (0)	3 (4.1)	
Stroke of other determined cause	2 (6.3)	0 (0)	2 (2.7)	
Patient Treated with Thrombolytic and/or Thrombectomy, *n* (%)	13 (40.6)	4 (9.5)	17 (23.0)	
IVT	9 (28.1)	4 (9.5)	13 (17.6)	**0.002**
EVT	3 (9.4)	0 (0)	3 (4.1)	
IVT and EVT	1 (3.1)	0 (0)	1 (1.4)	
Antiplatelet given at admission				
If yes, which one? *N* (%)	20 (62.5)	36 (85.7)	56 (75.7)	**0.021**
Aspirin	7/20 (35)	8/36 (22.2)	15/56 (26.8)	
Aspirin and Clopidogrel	12/20 (60)	26/36 (72.2)	38/56 (67.4)	
Aspirin and Ticagrelor	1/20 (5)	2/36 (5.6)	3 (5.4)	
NIHSS at the time of *intervention or ᐩ24h, median (IQR)	8.00 (6.75, 10.0)*	6.00 (5.00, 9.00)**ᐩ**	7.00 (5.25, 9.75)	0.0911
SBP at the time of *intervention or ᐩ24h, mean (SD)	158.00 (20.08)*	148.36 (23.92)**ᐩ**	152.53 (22.71)	0.064
DBP at the time of *intervention or ᐩ24h, mean (SD)	82.94 (17.64)*	80.38 (12.56)**ᐩ**	81.49 (14.91)	0.489
Presentation type: Rescue BT2 indication for Tirofiban initiation, *n* (%)				
Ineligible for reperfusion treatment and within 24 h after stroke onset	0 (0)	23 (54.8)	23 (31.1)	**<0.001**
Ineligible for reperfusion treatment and had progression 24-96 h after stroke onset	20 (62.5)	15 (35.7)	35 (47.3)	
Received IVT followed by END	7 (21.9)	2 (4.8)	9 (12.2)	
Received IVT followed by no improvement	2 (6.3)	2 (4.8)	4 (5.4)	
Other indications^	3 (9.3)	0 (0)	3 (3.9)	

^Other indications included starting Tirofiban for: P1 segment cardioembolic occlusion (2 patients, of which one later underwent EVT); microemboli on transcranial doppler following EVT, despite an improved NIHSS of 2 (1 patient). *intervention = tirofiban infusion or standard care. +24h = twenty four hours since admission.

mRS modified Rankin Scale; NIHSS, National Institutes of Health Stroke Scale; IVT, intravenous thrombolysis; SBP, systolic blood pressure; DBP, Diastolic blood pressure; EVT, Endovascular thrombectomy.

Categorical variables were compared between treatment groups using Pearson’s chi-square test or Fisher’s exact test, as appropriate. Continuous or ordinal variables were compared using the Mann–Whitney U test. Bold values indicate statistical significance with *p* value of <0.05.

Upon admission, patients presented with a median NIHSS of five and a median premorbid mRS of 0. The mean systolic blood pressure (SBP) on admission was 170.97 mmHg, and the mean diastolic blood pressure (DBP) was 93.81 mmHg. Regarding initial treatment, 23% of patients received IVT, and 75.7% received antiplatelet agents, with the most common regimen being dual antiplatelet therapy with aspirin and clopidogrel.

The baseline characteristics were generally comparable between the tirofiban and standard care groups. However, the standard care group had a statistically significant higher incidence of diabetes (69% vs. 15.6%; *p* < 0.001) and prior stroke (35.7% vs. 12.5%; *p* = 0.024), and had higher rate of treatment with antiplatelet therapy at admission (predominantly dual antiplatelet therapy with aspirin and clopidogrel) and a lower rate of IVT (9.5% vs. 31.3%) compared to the tirofiban group. Additionally, while there was no statistically significant difference in admission NIHSS for the tirofiban and the standard care group, the IQR for the tirofiban group was wider.

The primary stroke mechanism in both groups was small vessel disease (58.1%), followed by large artery disease (35.1%). Imaging frequently showed a left-sided cerebral infarct, often involving multiple areas, in both treatment arms. The second most common site of involvement differed between the groups, with the brainstem being the next most frequent site in the tirofiban group and cortical infarct in the standard care group.

Consistent with protocol-driven treatment selection, END was the predominant indication for tirofiban administration, occurring in 84.4% of patients. The cohort abided by the RESCUE BT2-definition of END in all cases. At the time of END, the median NIHSS score was 8, with mean SBP and DBP readings of 158 and 82.94, respectively. Operational metrics are detailed in Table 2. The median time from the last known well to the start of the tirofiban infusion was 28.8 h, and the infusion lasted for a median duration of 45.6 h. The median time from the order to the start of the tirofiban drip was 85 min, and 90.6% of patients received bolus dosing based on actual body weight.

**Table 2:** Safety, disposition, and operational feasibility outcomes.

Outcome	Tirofiban (*N* = 32)	Standard care (*N* = 42)	*p*-value
Safety outcomes			
Imaging done at 48 h after Tirofiban or after starting standard treatment, *n* (%)	28 (87.5)	12 (28.6)	**<0.001**
CT Head	25 (89.3)	8 (66.7)	
MRI Brain	3 (10.7)	4 (33.3)	
Any intracranial hemorrhage within 48 h after Tirofiban initiation, *n*/*N* (%)	0/28 (0)	0/12 (0)	—
sICH within 48 h after Tirofiban initiation by ECASS II, *n*/*N* (%)	0/28 (0)	0/12 (0)	—
sICH within 48 h after Tirofiban initiation by NINDS, *n*/*N* (%)	0/28 (0)	0/12 (0)	—
sICH within 48 h after Tirofiban initiation by SITS-MOST, *n*/*N* (%)	0/28 (0)	0/12 (0)	—
Extracranial hemorrhage, *n* (%)	3 (9.4)	0 (0)	0.077
Epistaxis	2 (6.3)		
Gastrointestinal Bleed	1 (3.1)		
Thrombocytopenia, *n* (%)	0 (0)	3 (4.1)	0.254
In-hospital mortality, *n* (%)	0 (0)	1 (2.4)	1.000
Discharge disposition, *n* (%)			
Inpatient Rehab	24 (75.0)	20 (47.6)	0.086
Home	5 (15.6)	7 (16.7)	
Skilled nursing facility	2 (6.2)	11 (26.2)	
Hospice	1 (3.1)	2 (4.8)	
Left AMA	0 (0)	1 (2.4)	
Expired	0 (0)	1 (2.4)	
Operational feasibility outcomes			
Time from last known normal to tirofiban initiation, hours, median (IQR)	28.8 (17.7,49.2)	—	—
Duration of tirofiban infusion, hours, median (IQR)	45.6 (41.4, 46.8)	—	—
Time from order to start of therapy, minutes, median (IQR)	85 (48.3, 111.3)	—	—
Actual body weight used for bolus dose, *n* (%)	29 (90.6)	—	—
Repeat imaging within 48 h from tirofiban initiation, *n* (%)	28 (87.5)	—	—
Deviation from tirofiban protocol, *n* (%)*	2 (6.25)	—	—

*Incorrect bolus or maintenance dose.

AMA, against medical advice; CT, computed tomography; ECASS II, European Cooperative Acute Stroke Study II; MRI, magnetic resonance imaging; NINDS, National Institute of Neurological Disorders and Stroke; sICH, symptomatic intracranial hemorrhage; SITS-MOST, Safe Implementation of Thrombolysis in Stroke-Monitoring Study. Bold values indicate statistical significance with *p* value of <0.05.

In the standard care group, patients were most often (54.8%) ineligible for reperfusion treatment and within 24 h after stroke onset. The next largest group (35.7%) consisted of patients experiencing stroke progression (END) between 24 and 96 h who were also ineligible for IVT.

### Safety and discharge disposition

In the tirofiban group, follow-up imaging was obtained in 87.5% of patients after completion of the infusion, which generally occurred within 48 h of treatment initiation. No patient in this group developed asymptomatic or symptomatic ICH, as defined by ECASS II, NINDS or SITS-MOST criteria (Table 2). No patient developed thrombocytopenia. The most common bleeding event was epistaxis, which occurred in two patients (6.3%): one case was chronic, and the other was mild and self-resolving. A single patient experienced a GI bleed attributed to a diverticular bleed on colonoscopy. One patient was discharged to hospice and passed away.

In contrast, follow-up imaging was performed in 28.6% of patients in the standard care group, and no hemorrhage, including sICH, was observed in those imaged. Three patients in the standard care group had pre-existing thrombocytopenia. One patient in the standard care group died due to an acute exacerbation of chronic respiratory failure.

Regarding discharge disposition (Table 2), a higher proportion of patients in the tirofiban group were discharged to inpatient rehabilitation. In contrast, patients in the standard-care group were most commonly discharged to inpatient rehabilitation or skilled nursing facilities. The percentage of patients discharged home was similar for both treatment groups. Compared with standard-care, tirofiban was associated with longer hospital length of stay in crude and baseline-adjusted analyses. However, this association did not remain statistically significant after additional adjustment for decision-point NIHSS.

### Discharge outcomes

Discharge outcomes are summarized in Table 3. Median discharge mRS was 4 in both groups, although the distribution was modestly shifted toward lower disability in the tirofiban group. In ordinal shift analysis, tirofiban was not significantly associated with discharge mRS in the unadjusted or baseline-adjusted models. After additional adjustment for decision-point NIHSS, tirofiban was associated with lower odds of a worse discharge mRS compared with standard care (OR 0.274, 95% CI 0.087–0.864; *p* = 0.027). In the propensity-matched sensitivity analysis, which included 27 patients in each group, the corresponding effect estimate was similar in magnitude but less precise and did not reach statistical significance.

**Table 3:** Discharge outcomes and sensitivity analyses.

Outcomes	Tirofiban	Standard care	Unadjusted estimate (95% CI)	*p*-value	Baseline-adjusted estimate (95% CI)*	*p*-value	Decision-point-adjusted estimate (95% CI)**	*p*-value
Unmatched discharge neurological outcome analyses								
Discharge NIHSS	5.00 (2.75, 8.00)	5.00 (5.00, 9.75)	−1.259 (−3.164, 0.646)	0.199	−0.388 (−2.513, 1.736)	0.721	−2.199 (−3.850, −0.548)	**0.011**
NIHSS change from 24 h/ END to discharge***	2 (0, 5)	0 (0, 1)	2.893 (1.526, 4.259)	**<0.001**	2.815 (1.173, 4.456)	**0.001**	2.815 (1.173, 4.456)	**0.001**
Hospital Length of Stay, days	6.00 (5.00, 7.00)	4.00 (2.25, 6.00)	1.954 (0.518, 3.390)	**0.009**	1.927 (0.063, 3.791)	**0.047**	1.272 (−0.614, 3.158)	0.191
Unmatched discharge functional outcome analysis								
Discharge mRS	4 (3, 4)	4 (3.25, 4.75)	0.477 (0.201, 1.133)	0.094	0.650 (0.228, 1.856)	0.421	0.274 (0.087, 0.864)	**0.027**
Discharge mRS 0–3, *n* (%)	13 (40.6)	11 (26.2)	1.928 (0.720, 5.165)	0.192	1.512 (0.436, 5.247)	0.515	2.680 (0.653, 10.989)	0.171
Propensity-matched analysis								
Discharge mRS, median (IQR)	4 (3, 4)	4 (3, 4.5)	0.511 (0.191, 1.364)	0.180	0.699 (0.214, 2.282)	0.552	0.276 (0.076, 1.004)	0.051
Discharge mRS 0–3, *n* (%)	12 (44.4)	8 (29.6)	1.900 (0.619, 5.834)	0.262	1.318 (0.327, 5.316)	0.698	2.960 (0.554, 15.822)	0.204
Sensitivity analysis after excluding patients with protocol deviation								
Discharge mRS, median (IQR)	4 (3, 4)	4 (3.25, 4.75)	0.513 (0.207, 1.272)	0.150	0.985 (0.942, 1.030)	0.367	0.244 (0.064, 0.934)	**0.039**
Discharge mRS 0–3, *n* (%)	10 (37.0)	11 (26.2)	1.658 (0.586, 4.694)	0.341	1.724 (0.432, 6.880)	0.441	2.794 (0.570, 13.690)	0.205

CI, confidence interval; NIHSS, National Institutes of Health Stroke Scale; END, early neurological deterioration; mRS, modified Rankin Scale; OR, odds ratio.

For ordinal discharge mRS, estimates are ORs from ordinal logistic shift analysis; OR <1 indicates lower odds of worse discharge mRS. For discharge mRS 0–3, estimates are odds ratios; OR >1 indicates higher odds of mRS 0–3. For discharge NIHSS, NIHSS change, and hospital length of stay, estimates are linear regression coefficients (*β*) comparing tirofiban with standard care. Negative *β* values indicate lower values in the tirofiban group; for NIHSS change, positive *β* values indicate greater improvement.

Propensity score matching was performed using age, sex, and premorbid mRS. ORs were estimated using univariable or multivariable logistic regression, as appropriate. Continuous outcomes were analyzed using linear regression. *Baseline-adjusted models were adjusted for age, sex, NIHSS at admission, prior stroke, diabetes mellitus, and thrombolytic use. **Decision-point-adjusted models additionally included NIHSS at END/24 h, except when the NIHSS measure was embedded in the outcome definition. ***NIHSS-derived outcomes were not adjusted for NIHSS measures that are embedded in the outcome to avoid redundant adjustment and preserve interpretability. Bold values indicate statistical significance with *p* value of <0.05.

For the binary analysis of discharge mRS 0–3, estimates favored tirofiban in both unmatched and propensity-matched analyses, but confidence intervals were wide and none of these comparisons reached statistical significance.

The median discharge NIHSS was similar between groups, but decision-point adjusted regression estimated significantly lower NIHSS at discharge in the tirofiban group compared to the standard-care group (*β* = −2.199, 95% CI -3.850 to −0.548; *p* = 0.011). Furthermore, adjusted regression estimated greater improvement in NIHSS score from END/24 h to discharge in the tirofiban group (*β* = 2.815, 95% CI 1.173 to 4.456; *p* = 0.001).

The 90-day mRS score was available for 22 out of 32 patients (68.75%) in the tirofiban group and 12 out of 42 patients (28.57%) in the standard-care group, indicating substantial differential missingness. Little’s MCAR test did not detect statistically significant evidence against the assumption that data were missing completely at random (*p* = 0.385). Among patients with available follow-up, median mRS at 90 days was 3 in the tirofiban group and 3.5 in the standard-care group. Analyses of 90-day mRS are summarized in Supplementary Table 2. Complete-case analyses did not demonstrate statistically significant differences in ordinal 90-day mRS or binary 90-day mRS 0–3 before or after adjustment. In multiple-imputation analysis using 50 imputed datasets, decision-point adjusted ordinal shift analysis favored tirofiban (OR 0.251, 95% CI 0.065–0.966; *p* = 0.045), whereas binary 90-day mRS 0–3 was not associated after adjustment. In general, the associations were consistent between multiple imputation and complete-case (non-imputed) analyses across both unadjusted and decision-point–adjusted models. Propensity-matched analyses similarly did not demonstrate statistically significant differences. In general, the associations were consistent before and after propensity matching. However, given the extent of missingness, these analyses were considered exploratory.

Sensitivity analyses excluding patients with documented protocol deviations were performed for discharge mRS and 90-day mRS outcomes (Table 3; Supplementary Table 2). After excluding protocol-non-adherent patients, decision-point-adjusted ordinal shift analysis of discharge mRS remained associated with lower odds of worse discharge mRS in the tirofiban group compared with standard care (OR 0.244, 95% CI 0.064–0.934; *p* = 0.039). For discharge mRS 0–3, estimates remained statistically nonsignificant. For 90-day mRS, exclusion of protocol-non-adherent patients did not demonstrate statistically significant differences in either ordinal mRS or mRS 0–3. Overall, the protocol-adherence sensitivity analysis did not suggest a quantitatively different pattern from the primary mRS analyses, but interpretation remained limited by reduced sample size and wide confidence intervals.

## Discussion

In this single-center retrospective study, we evaluated the use of an IV tirofiban protocol based on the RESCUE BT2 trial at a contemporary U.S. comprehensive stroke center. To our knowledge, this is the first study to assess the safety, operational feasibility, and exploratory clinical outcomes of intravenous tirofiban in a U.S. population meeting RESCUE BT2 eligibility criteria.

The principal findings were threefold. First, no sICH, asymptomatic ICH, or thrombocytopenia occurred among tirofiban-treated patients, supporting the safety of this approach in a carefully selected U.S. population. Second, tirofiban administration was operationally feasible, with rapid treatment initiation, relatively high protocol adherence, and consistent follow-up imaging. Third, although exploratory and hypothesis-generating, adjusted analyses suggested improved neurological and functional outcomes at discharge among tirofiban-treated patients compared to the historical standard treatment group.

These findings are notable given that nearly all contemporary randomized trials evaluating tirofiban in AIS, including RESCUE BT2 and TREND, have been conducted in predominantly Han Chinese populations. In these cohorts, intracranial atherosclerotic disease is substantially more prevalent than in North American stroke populations, raising important questions about external validity. Furthermore, differences in stroke systems of care, imaging practices, antithrombotic strategies, and patient demographics may influence treatment effects. Our cohort was predominantly composed of patients with small-vessel disease and large-artery atherosclerosis treated within a real-world comprehensive stroke workflow, providing important preliminary evidence that tirofiban can be integrated safely outside the populations in which it has primarily been studied.

Safety was the primary objective of this study. Among patients treated with tirofiban, no cases of sICH occurred according to ECASS II, NINDS, or SITS-MOST definitions, and no patient developed thrombocytopenia. Extracranial bleeding events were uncommon and generally minor, consisting of two episodes of epistaxis and one gastrointestinal bleed attributed to diverticular disease. Although the sample size was insufficient to exclude uncommon adverse events, the observed safety profile is consistent with prior randomized trials and recent meta-analyses demonstrating no significant increase in sICH or mortality with tirofiban compared with standard medical therapy (22–31). These findings are particularly reassuring given that many patients in the tirofiban cohort experienced neurological worsening before treatment initiation, representing a clinically vulnerable population often perceived to be at increased hemorrhagic risk.

From an implementation perspective, our findings support the operational feasibility of integrating tirofiban into routine stroke care. Median time from order entry to infusion initiation was 85 min, weight-based dosing adherence exceeded 90%, and protocol-directed follow-up imaging was obtained in nearly all treated patients. These metrics suggest that IV tirofiban can be incorporated into existing comprehensive stroke workflows without substantial disruption. At the same time, protocol deviations occurred in approximately 15.6% of treated patients, underscoring the practical challenges of translating highly selective randomized trial protocols into real-world practice particularly when stroke mechanism, timing, and neurological trajectory evolve dynamically.

While the study was not powered to establish efficacy, several exploratory findings warrant consideration. After adjustment for baseline characteristics and neurological severity immediately preceding treatment, tirofiban was associated with a favorable shift in discharge mRS and lower discharge NIHSS scores. Patients receiving tirofiban also demonstrated greater neurological improvement between the treatment decision point (END or 24-h assessment) and discharge. These findings remained directionally consistent in propensity-matched and protocol-adherent sensitivity analyses, although statistical significance was not uniformly maintained, likely reflecting limited sample size and reduced precision. Accordingly, these observations should be viewed as hypothesis-generating rather than definitive evidence of treatment benefit.

Our findings are broadly consistent with the growing literature evaluating tirofiban in AIS. A recent meta-analysis by Monteiro et al. demonstrated improved functional outcomes and favorable ordinal mRS shifts at 90 days without increased hemorrhagic complications or mortality (31). Similarly, the ESCAPIST trial reported improved early neurological recovery among patients treated with tirofiban (29). Although our primary functional endpoint was discharge rather than 90-day outcome, the observed association between tirofiban and improved discharge neurological status is directionally concordant with these prior studies. The lack of statistically significant differences in several binary outcome analyses, including discharge mRS 0–3, likely reflects limited power and the inherent loss of information associated with dichotomizing functional outcomes.

An important and potentially novel aspect of this study is the large proportion of patients treated for END. More than 80% of patients in the tirofiban cohort experienced END before treatment initiation, compared with approximately 40% of participants in RESCUE BT2 (28). END remains one of the strongest predictors of poor functional outcome after ischemic stroke, yet targeted treatment options remain limited. While accelerated platelet aggregation and microvascular thrombosis have been proposed as contributors to END, few interventions have specifically been evaluated after deterioration has already occurred. In contrast to TREND, which primarily evaluated tirofiban as a strategy to prevent neurological deterioration (27), our cohort largely reflects treatment after clinical worsening had already developed. Although causal inference cannot be established from this observational study, the favorable adjusted outcomes observed despite the higher prevalence of END suggest that this population may warrant focused investigation in future prospective studies.

The NIHSS findings merit careful interpretation. We did not observe a significant difference in change from admission NIHSS to discharge NIHSS after adjustment. However, patients treated with tirofiban had significantly lower discharge NIHSS scores and greater improvement from the decision point (END or 24-h assessment) to discharge. One explanation is the greater variability in baseline stroke severity within the tirofiban cohort. Although median admission NIHSS scores were similar between groups, the tirofiban group demonstrated a wider distribution of baseline severity, which may have reduced the ability to detect differences using admission-to-discharge change scores. Analyses anchored to the treatment decision point may better reflect the clinical effect of tirofiban because they account for neurological status immediately before treatment initiation.

In contrast to the historical standard care group, the most common trigger for tirofiban use in our study was END. Consequently, patients in the tirofiban group were substantially more likely to have experienced END pre-treatment than those in the standard care group (84.3% vs. 40.4%). While this protocol-driven treatment design may introduce confounding by indication, the observed difference in END rates reflects the criteria for tirofiban use rather than a baseline imbalance between groups.

Interpretation of the 90-day outcomes requires greater caution. Follow-up mRS data were incomplete and disproportionately missing in the standard-care cohort. Although Little’s MCAR test did not identify evidence against missing completely at random assumptions, statistical power was limited and differential follow-up remains a significant concern. Complete-case and propensity-matched analyses did not demonstrate statistically significant differences, whereas on multiple-imputation analysis, decision-point adjusted ordinal shift analysis favored tirofiban. This is likely because the imputation had larger data. However, given the extent of missingness, these findings should be considered exploratory and insufficient to draw definitive conclusions regarding long-term functional recovery.

Compared with the RESCUE BT2 trial, the median time from last known well to tirofiban initiation was markedly longer in our cohort (29 h vs. 11 h). This is possibly because tirofiban was most often initiated as a rescue therapy for END in our cohort. Given that END commonly develops within the first 24–72 h after stroke onset, a longer interval to treatment would be expected in this setting (40–42). Accordingly, the timing of tirofiban administration in our cohort may better represent real-world practice patterns for selected patients with delayed progression or END rather than broader AIS populations.

Our study has several important limitations. First, its retrospective, non-randomized design introduces the possibility of residual confounding and treatment-selection bias despite multivariable adjustment and propensity-score matching. Second, the small sample size restricts statistical power, particularly for uncommon safety events and binary functional outcomes, and nonsignificant findings should not be interpreted as equivalence. Third, use of a historical control group introduces potential secular confounding related to evolving stroke workflows and care practices. Fourth, the inability to identify all potentially eligible untreated patients limits assessment of treatment uptake and selection processes. Fifth, differential missingness in 90-day follow-up substantially limits interpretation of long-term outcomes. Sixth, provider discretion in treatment initiation and heterogeneity in timing relative to symptom onset and prior therapies introduce additional complexity. Finally, the standard-care cohort underwent less frequent follow-up neuroimaging, potentially limiting direct comparison of asymptomatic hemorrhagic events.

In conclusion, our study provides preliminary real-world evidence that IV tirofiban can be implemented safely within a U. S. comprehensive stroke center for carefully selected patients with AIS who meet RESCUE BT2–type eligibility criteria. While the observational design and limited sample size precludes definitive conclusions regarding efficacy, the consistency of the observed neurological and functional signals across multiple adjusted and sensitivity analyses, coupled with a favorable safety profile, suggests that tirofiban may represent a promising therapeutic option for selected patients with AIS who are ineligible for reperfusion therapies or who experience END. These results support further evaluation through multicenter prospective registries and randomized controlled trials in diverse populations, with particular attention to patients experiencing END. Future studies should clarify optimal patient selection, treatment timing, and comparative effectiveness relative to contemporary antiplatelet strategies, including dual antiplatelet therapy, to determine whether targeted inhibition of platelet aggregation can address the current therapeutic gap for patients with AIS and END.

## Data Availability

The original contributions presented in the study are included in the article/Supplementary material, further inquiries can be directed to the corresponding author.
