# Supplementary material for: Real-world experience with RESCUE BT2 tirofiban protocol versus standard care for select patients with acute ischemic stroke at a United States comprehensive stroke center: a single-center retrospective study
Source: Front Neurol. 2026 Jul 22;17:1768603. doi: 10.3389/fneur.2026.1768603 (PMC13437403; doi:10.3389/fneur.2026.1768603)
Supplement: Supplementary file 1 [file Table_1.docx]

**Supplementary tables with figure legends**

**Supplementary Table 1: Standardized mean difference after Age, Sex, and premorbid mRS propensity matching**

**Summary statistics**

| Variable | All data (Before matching) | | | Matched data | | |
| --- | --- | --- | --- | --- | --- | --- |
|  | Means Treated | Means Control | Std. Mean Diff. | Means Treated | Means Control | Std. Mean Diff. |
| distance | 0.4596 | 0.4117 | 0.5996 | 0.4718 | 0.4691 | 0.0335 |
| Age | 67.2813 | 65.8571 | 0.1392 | 67.2593 | 67.2222 | 0.0036 |
| sexFemale | 0.3125 | 0.4048 | -0.1990 | 0.3333 | 0.3704 | -0.0799 |
| sexMale | 0.6875 | 0.5952 | 0.1990 | 0.6667 | 0.6296 | 0.0799 |
| mRS_premorbid | 0.2500 | 0.7381 | -0.8594 | 0.1111 | 0.1111 | 0.0000 |

**Sample sizes**

|  | Control | Treated |
| --- | --- | --- |
| All | 42 | 32 |
| Matched | 27 | 27 |
| Unmatched | 15 | 5 |
| Discarded | 0 | 0 |
