# Supplementary material for: Real-world experience with RESCUE BT2 tirofiban protocol versus standard care for select patients with acute ischemic stroke at a United States comprehensive stroke center: a single-center retrospective study
Source: Front Neurol. 2026 Jul 22;17:1768603. doi: 10.3389/fneur.2026.1768603 (PMC13437403; doi:10.3389/fneur.2026.1768603)
Supplement: Supplementary file 2 [file Table_2.docx]

**Supplementary Table 2: 90-day mRS complete-case, multiple-imputation, propensity-matched sensitivity analyses, and sensitivity analyses after excluding patients with protocol deviation.**

| **Outcome** | **Tirofiban** | **Standard Care** | **Unadjusted OR (95% CI)** | **p-value** | ***Baseline-adjusted OR (95% CI)** | **p-value** | ****Decision-point-adjusted OR (95% CI)** | **p-value** |
| --- | --- | --- | --- | --- | --- | --- | --- | --- |
| **mRS at 90-days available, % (n/N)** | 68.75 (22/32) | 28.57 (12/42) |  |  |  |  |  |  |
| **Complete-case analysis** | | | | | | | | |
| **mRS at 90 days, median (IQR)** | 3.0 (1.00, 4.00) | 3.5 (3.0, 4.25) | 0.414 (0.116, 1.476) | 0.174 | 1.091 (0.213, 5.595) | 0.917 | 0.295 (0.040, 2.162) | 0.230 |
| **mRS 0–3 at 90 days, n (%)** | 14 (66.7) | 6 (50.0) | 2.000 (0.469, 8.530) | 0.349 | 0.388 (0.042, 3.527) | 0.400 | 0.607 (0.041, 8.923) | 0.716 |
| **Multiple-imputation (m=50)** | | | | | | | | |
| **mRS at 90 days, median (IQR)** | 2.94 (0.68, 3.74) | 3.28 (2.46, 4.54) | 0.411 (0.156, 1.084) | 0.071 | 0.586 (0.181, 1.894) | 0.364 | 0.251 (0.065, 0.966) | **0.0447** |
| **mRS 0–3 at 90day, n (%)** | 22.9 (71.7) | 22.4 (53.2) | 2.230 (0.718, 6.929) | 0.162 | 1.352 (0.283, 6.467) | 0.700 | 2.609 (0.429, 15.864) | 0.2902 |
| **Propensity-matched analysis** | | | | | | | | |
| **mRS at 90 days, median (IQR)** | 3 (0.25, 4.0) | 3 (3.0, 4.5) | 0.393 (0.085, 1.825) | 0.233 | 0.841 (0.135, 5.222) | 0.852 | 0.246 (0.025, 2.475) | 0.234 |
| **mRS 0–3 at 90 days, n (%)** | 12 (66.7) | 4 (57.1) | 1.500 (0.251, 8.977) | 0.657 | 0.497 (0.046, 5.411) | 0.566 | 0.447 (0.019, 10.339) | 0.615 |
| **Sensitivity analysis after excluding patients with protocol deviations** | | | | | | | | |
| **mRS at 90 days, median (IQR)** | 3 (1.5, 4.00) | 3.5 (3.0, 4.25) | 0.418 (0.110, 1.587) | 0.199 | 0.538 (0.093, 3.112) | 0.489 | 0.145 (0.015, 1.438) | 0.099 |
| **mRS 0–3 at 90 days, n (%)** | 12 (66.7) | 6 (50.0) | 2.000 (0.448, 8.936) | 0.364 | 0.477 (0.051, 4.459) | 0.516 | 0.718 (0.044, 11.681) | 0.816 |

Abbreviations: *CI, confidence interval; OR, odds ratio; mRS, modified Rankin Scale; NIHSS, National Institutes of Health Stroke Scale; END, early neurological deterioration.*

*For ordinal 90-day mRS, OR <1 indicates lower odds of worse 90-day mRS. For 90-day mRS 0–3, OR >1 indicates higher odds of mRS 0–3.*

*Complete-case analyses included patients with available 90-day mRS data. Multiple-imputation analyses were performed using 50 imputed datasets. Propensity score matching was performed using age, sex, and premorbid mRS.*

*ORs were estimated using ordinal logistic regression for mRS shift analyses and logistic regression for binary mRS 0–3 analyses. *Baseline-adjusted models were adjusted for age, sex, NIHSS at admission, prior stroke, diabetes mellitus, and thrombolytic use. **Decision-point-adjusted models additionally included NIHSS at END/24h.*

*Bold p values indicate statistical significance with p value <0.05.*
